# Usefulness of speckle-tracking echocardiography for early detection in children with Duchenne muscular dystrophy: a meta-analysis and trial sequential analysis

**DOI:** 10.1186/s12947-020-00209-y

**Published:** 2020-07-10

**Authors:** Guang Song, Jing Zhang, Xin Wang, Xintong Zhang, Feifei Sun, Xiaona Yu

**Affiliations:** Department of Ultrasound, Shengjing Hospital of China Medical University, 36 Sanhao St, Heping District, Shenyang, 110001 Liaoning Province China

**Keywords:** Cardiomyopathy, Duchenne muscular dystrophy, Pediatrics, Speckle-tracking echocardiography, Strain

## Abstract

**Background:**

Duchenne muscular dystrophy (DMD) is the most common form of inherited muscle disease in children. The incidence of cardiomyopathy induced by DMD increases with age. Left ventricular ejection fraction usually fails to reflect the subclinical left ventricular dysfunction. Several studies have assessed this dysfunction using myocardial strain measured by speckle-tracking echocardiography (STE). However, the results were inconsistent and incomplete.

**Methods:**

Several databases were searched from their inception to February 5, 2020. The summarized weighted mean difference (WMD) with 95% confidence intervals (CIs) were estimated for myocardial strain between DMD and healthy controls and a meta-analysis was conducted. Trial sequential analysis estimated whether the resulting evidence was sufficient.

**Results:**

Eight studies with a total of 269 DMD children and 299 healthy participants were included. STE revealed that global longitudinal strain (GLS), global circumferential strain, average longitudinal strain (measured by two-dimensional STE at the apical four-chamber view), and average circumferential strain (measured by two-dimensional STE at the papillary muscle short-axis level) decreased (WMD = 4.17, 95% CI: 3.03–5.32; WMD = 3.98, 95% CI: 0.29–7.68; WMD = 4.18, 95% CI: 2.75–5.62; and WMD = 4.90, 95% CI: 2.38–7.43, respectively; all *P* < 0.05) compared with the controls and global radial strain was unchanged in the DMD group (WMD = − 4.33, 95% CI: − 9.53–0.87, *P* = 0.103). Trial sequential analysis indicated that available GLS samples were sufficient and confirmed that adequate evidence was accumulated. The credibility of other myocardial strains was questioned due to insufficiently involved studies.

**Conclusion:**

GLS can be useful for early detection of left ventricle myocardial dysfunction in children with DMD.

## Introduction

Duchenne muscular dystrophy (DMD) is an X-linked recessive disorder caused by the lack of dystrophin encoded by the *DMD* gene. It is the most common form of inherited muscle disease in children [1]. DMD is a severe disease with muscular dystrophy that begins at the age of about 7 years and then rapidly and progressively leads to a loss of independent ambulation by the age of 12 years, followed by scoliosis, loss of upper limb function, respiratory insufficiency, or cardiomyopathy [2].

The incidence of DMD-associated cardiomyopathy increases with age, affecting 30% of 14-year-olds, 50% of 18-year-old, and all older patients [3]. However, cardiac dysfunction in DMD children has not been treated seriously enough because more than 30% of DMD patients have not undergone echocardiography examinations [4]. Recent guidelines have suggested that cardiac imaging should be performed every 2 years (since diagnosis to 10 years of age) or annually (from 10 to 20 years of age) and recommended that echocardiography should be routinely used in the screening and follow-up care of DMD patients [5, 6].

Echocardiography is the most commonly used imaging modality to assess cardiac function. Previous animal studies have usually used echocardiography to assess cardiac function. They revealed that some strain parameters were significantly changed in the DMD dog/mouse model and that strain analysis using speckle-tracking echocardiography (STE) is a feasible and sensitive approach for detecting cardiac dysfunction [7, 8]. STE can quantify myocardial strain and detect subclinical left ventricular dysfunction before left ventricular ejection fraction (LVEF) begins decreasing [9]. Previous STE studies have revealed altered myocardial strain in DMD children with normal fractional shortening and LVEF [10, 11]. Since then, an increasing number of STE studies have focused on evaluating myocardial strain in DMD children. However, some results of these studies were inconsistent and incomplete and might have limited statistical power because individual studies have relatively small sample sizes and analyze partial myocardial strain parameters. Therefore, meta-analysis aiming to provide a more comprehensive summary to evaluate the value of myocardial strain in children with DMD was carried out in the present study.

## Methods

### Search strategy

Two investigators (GS and JZ) independently searched PubMed, Web of Science, EMBASE, and Google scholar databases from their inception to February 5, 2020 to identify relevant studies. The following search keywords were included: ‘Duchenne Muscular Dystrophy’, ‘echocardiography’, and ‘strain’. This study only focused on human studies.

### Study selection and exclusion

Original studies were eligible if the following criteria were met: (i) observational study; (ii) the study investigated myocardial strain in DMD children compared to healthy participants; (iii) myocardial strain was measured by STE; and (iv) global longitudinal strain (GLS), global circumferential strain (GCS), and global radial strain (GRS) were defined as the global strain from the left ventricular 16/17-segment model. Average longitudinal strain (LS) was measured using two-dimensional STE at the apical four-chamber view and average circumferential strain (CS) was measured by two-dimensional STE at the papillary muscle short-axis level [12]. LVEF was measured using the biplane modified Simpson’s method.

Original studies were ineligible if the following criteria were met: (i) reviews, letters, or case reports; (ii) DMD children with LVEF < 45% or fractional shortening < 28%; (iii) invalid analysis, or did not report the data necessary for calculating the mean and standard deviation of myocardial strain; or (iv) did not include a healthy control group. If there were several publications from the same study, the study with the most cases and relevant information was included.

### Data extraction and quality assessment

Data extraction was performed independently by two of the reviewers (GS and JZ). Disagreements were discussed and resolved by consensus or by involving a third reviewer (XW) for adjudication. The extracted clinical data included the first author, year of publication, case number in the DMD and control groups, age, weight, height, heart rate, and country. The extracted echocardiographic data included LVEF, GLS, GCS, GRS, LS, and CS. This study did not contain the analysis of the average radial myocardial strain (from papillary muscle short-axis level) because that radial myocardial strain is not comparable among different ultrasound machines and software packages [13, 14].

The Newcastle–Ottawa Scale (NOS) was used to assess the quality of all included studies. The NOS quality score was evaluated as follows: ≤ 5, low quality; 6–7, medium quality; and 8–9, high quality. Two authors (XW and XZ) independently assessed the quality of the included studies. Disagreements were resolved by discussion.

### Statistical analysis

The pooled effects were presented as the weighted mean difference (WMD) with 95% confidence intervals (CIs). Heterogeneity was assessed using the *I*^2^ statistic. If heterogeneity was not present (*P* > 0.1 or *I*^2^ < 50%), a fixed-effects model was used to estimate the pooled WMD. Otherwise, a random-effects model was utilized. Subgroup stratified analysis was performed using country (“USA/Europe/Egypt” vs. “China/Korea”), quality of study (“medium quality” vs. “high quality”), method (“two-dimensional STE” vs. “three-dimensional STE”), and machine/software (“GE Vivid E9 with EchoPAC” vs. “Phillps iE33 with Qlab”) parameters. Sensitivity analyses were directed to assess the influence of the individual study on the overall estimate. Study effects such as publication bias were evaluated using Egger’s tests and a *p*-value < 0.1 was considered statistically significant for asymmetry. Statistical analyses were performed using Stata (version 14.0; StataCorp, College Station, TX, USA).

### Trial sequential analysis (TSA)

TSA was conducted to maintain a 95% CI, 20% relative risk reduction, 5% level of type I error and 20% level of type II error (a power of 80%). When the cumulative Z-curve crossed the trial sequential monitoring boundary or exceeded the required information size line, it was considered to be an indicator of sufficient and firm evidence, with no further studies required. Otherwise, additional studies were needed. Trial Sequential Analysis (version 0.9.5.10 Beta) was used in this study.

## Results

### Description of the included studies

**Fig. 1:**
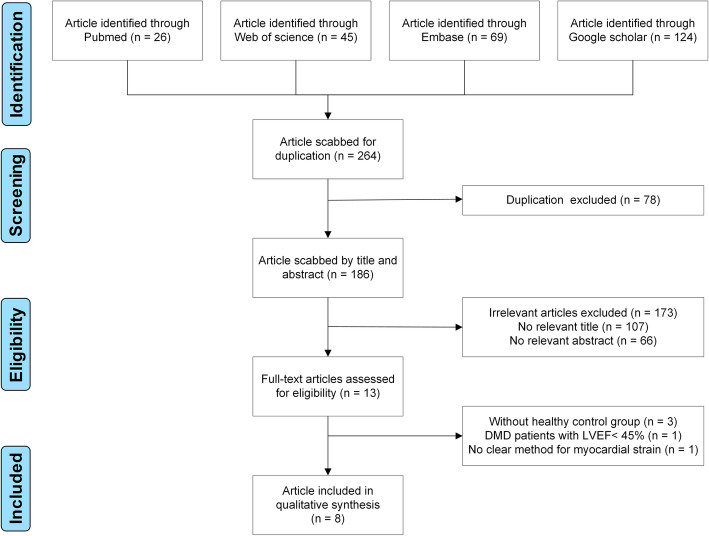
Flow-chart of study selection

A total of 264 potentially relevant publications from four databases, including 26 from PubMed, 69 from Embase, 45 from Web of Science, and 124 from Google scholar (Fig. 1) were identified and reviewed. After application of the inclusion and exclusion criteria, eight observational studies were identified [10, 11, 15–20].

The baseline characteristics of the eight included studies are shown in Table 1. These studies were published between 2013 and 2019. In total, 269 DMD children and 299 healthy participants were included. Male children were the main subjects of four studies. DMD children had a mean age of 9.34 ± 3.62 years, compared to 10.45 ± 5.88 years in the control group. Four studies were conducted in USA/Europe/Egypt and included predominantly Caucasian patients. The other four studies were conducted in China/Korea, which are mainly East Asian. The NOS score ranged from 7 to 9, indicating that low-quality studies were not involved.

**Table 1:** Characteristics of included studies

First author	Year	Number of cases (DMD/control)	Male (DMD/control, %)	Age (year)		Weight (DMD/control, kg)	Height (DMD/control, cm)	Heart rate (DMD/control, bpm)	Country	NOS score
				DMD	Control					
Ryan	2013	63/61	100%/100%	5.6 ± 0.2	5.2 ± 0.2	19.7/21.0	106.7/111.4	100.8/87.7	USA	9
Spurney	2015	35/33	NR	13.0 ± 2.0	13.0 ± 3.0	NR	NR	92.0/67.0	USA	8
Taqatqa	2016	19/16	100%/100%	11.0 ± 3.7	12.6 ± 3.1	38.0/54.4	136.0/164.0	98/69	USA	7
Cho	2018	13/26	NR	9.7 ± 2.2	9.7 ± 2.2	NR	NR	91/80	Korea	7
Yu	2019	56/31	NR	8.8 ± 1.9	8.4 ± 1.8	27.9/28.9	128.6/132.1	NR	China	8
Wang	2019	30/30	NR	7.8 ± 1.8	8.3 ± 1.7	27.7/28.1	122.6/126.6	89/91	China	6
Amedro	2019	36/72	100%/100%	11.0 ± 3.8	10.0 ± 3.5	36.8/37.9	134.9/143.7	96/75	Europe	9
Habib	2019	17/30	82%/83%	14.5 ± 3.7	22.3 ± 9.5	55.4/54.9	110.0/146.0	92/84	Egypt	6

*DMD* Duchenne muscular dystrophy, *NOS* Newcastle–Ottawa Scale, *NR* No reported

Measurement of GLS, GCS, LS, and CS using 2D STE was feasible in all participants of the control group. 100% (85/85) of GLS, 100% (19/19) of GCS, 94% (49/52) of LS, and 96% of (145/151) of CS could be measured by 2D STE in DMD patients. Measurement of GLS, GCS, and GRS using 3D STE was feasible in all subjects of the DMD and control groups. Five studies contained the intraobserver and interobserver variability test for myocardial strain [10, 11, 16–18]. The intraobserver and interobserver variability values were low in those five studies.

### The difference in myocardial strain between DMD and control groups

**Fig. 2:**
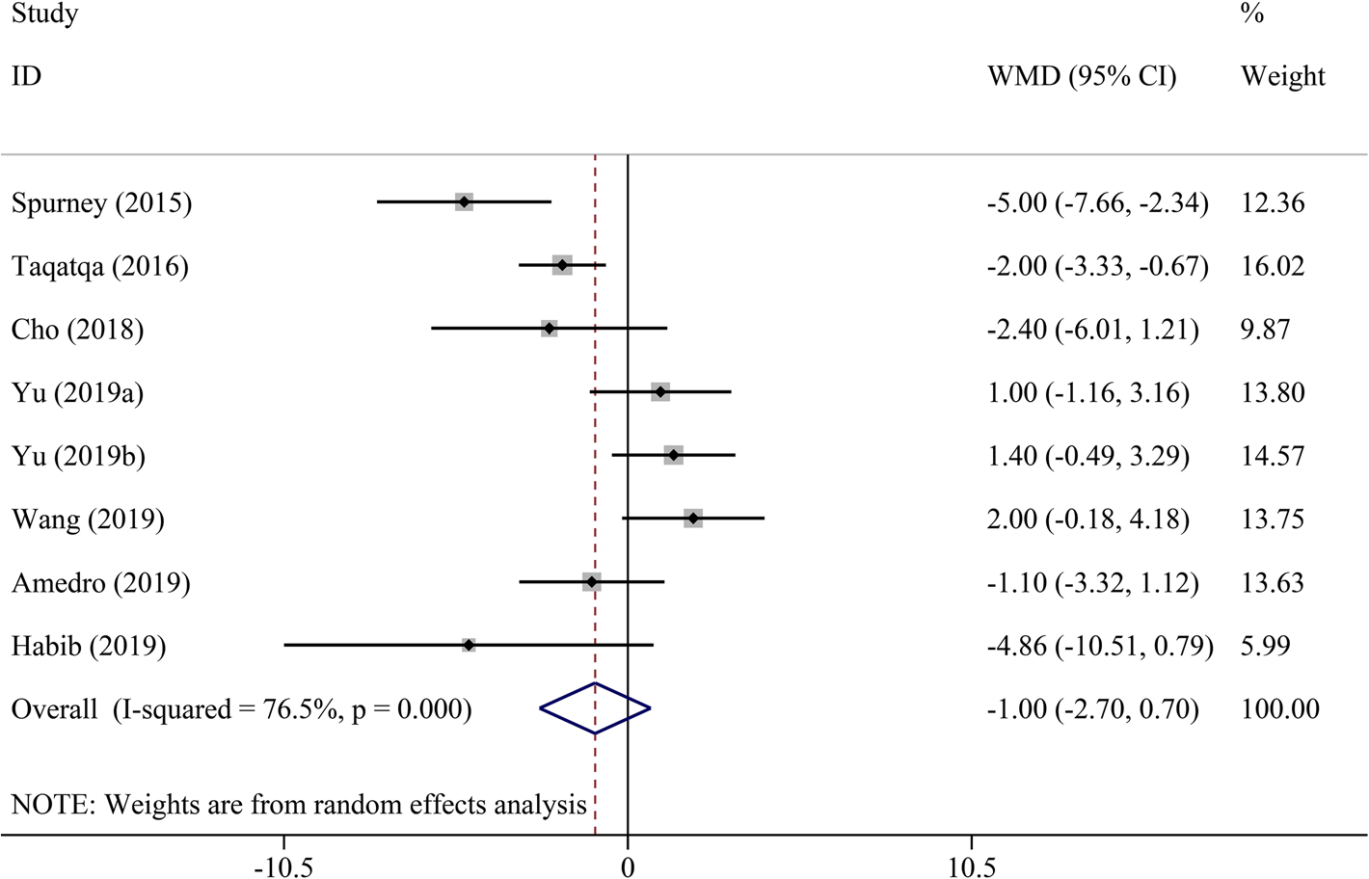
The meta-analysis of WMD in LVEF between DMD and control groups. CI, confidence interval; DMD, Duchenne muscular dystrophy; LVEF, left ventricular ejection fraction; WMD, weighted mean difference

**Fig. 3:**
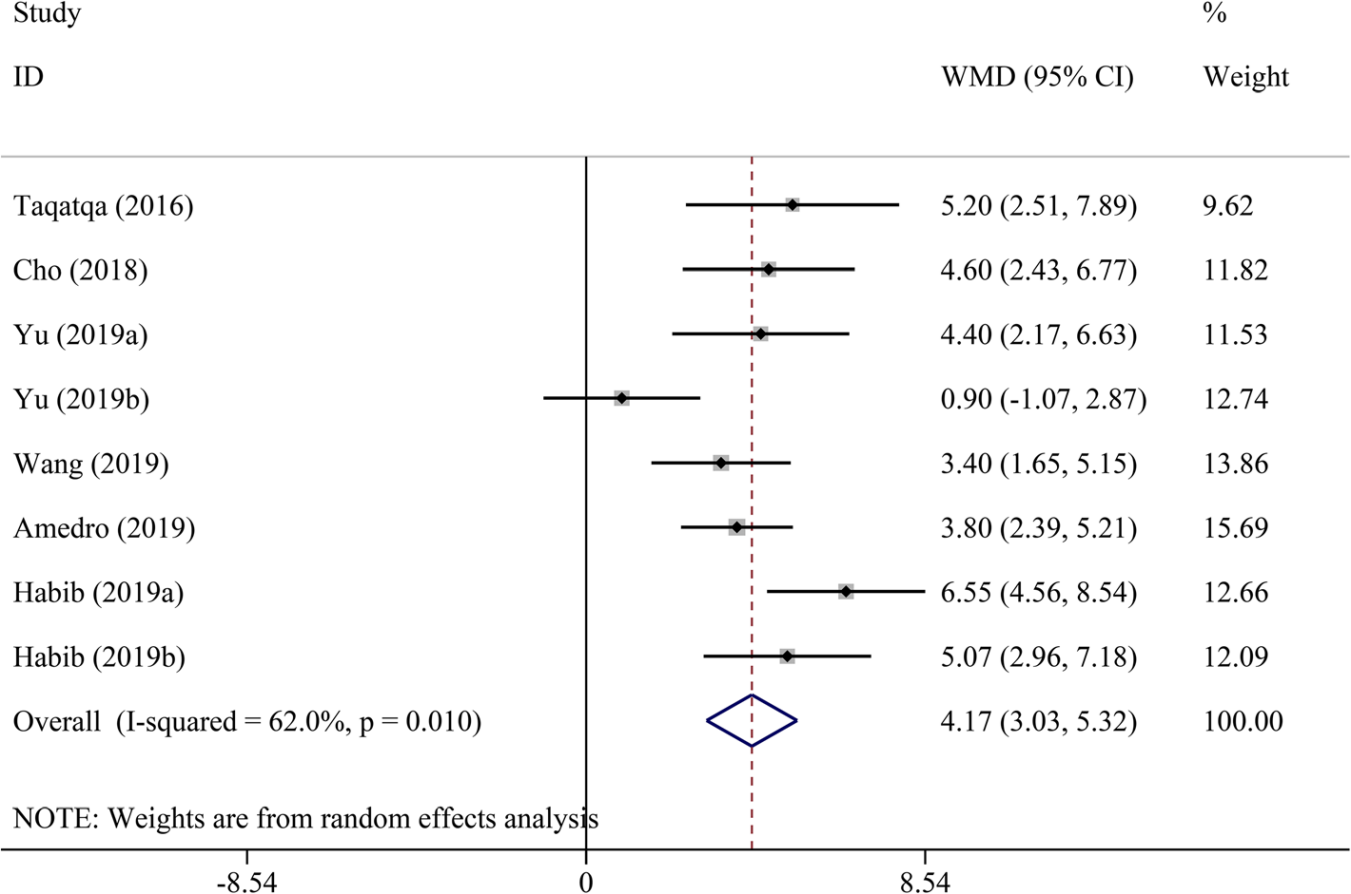
The meta-analysis of WMD in GLS between DMD and control groups. CI, confidence interval; DMD, Duchenne muscular dystrophy; GLS, global longitudinal strain; WMD, weighted mean difference

**Fig. 4:**
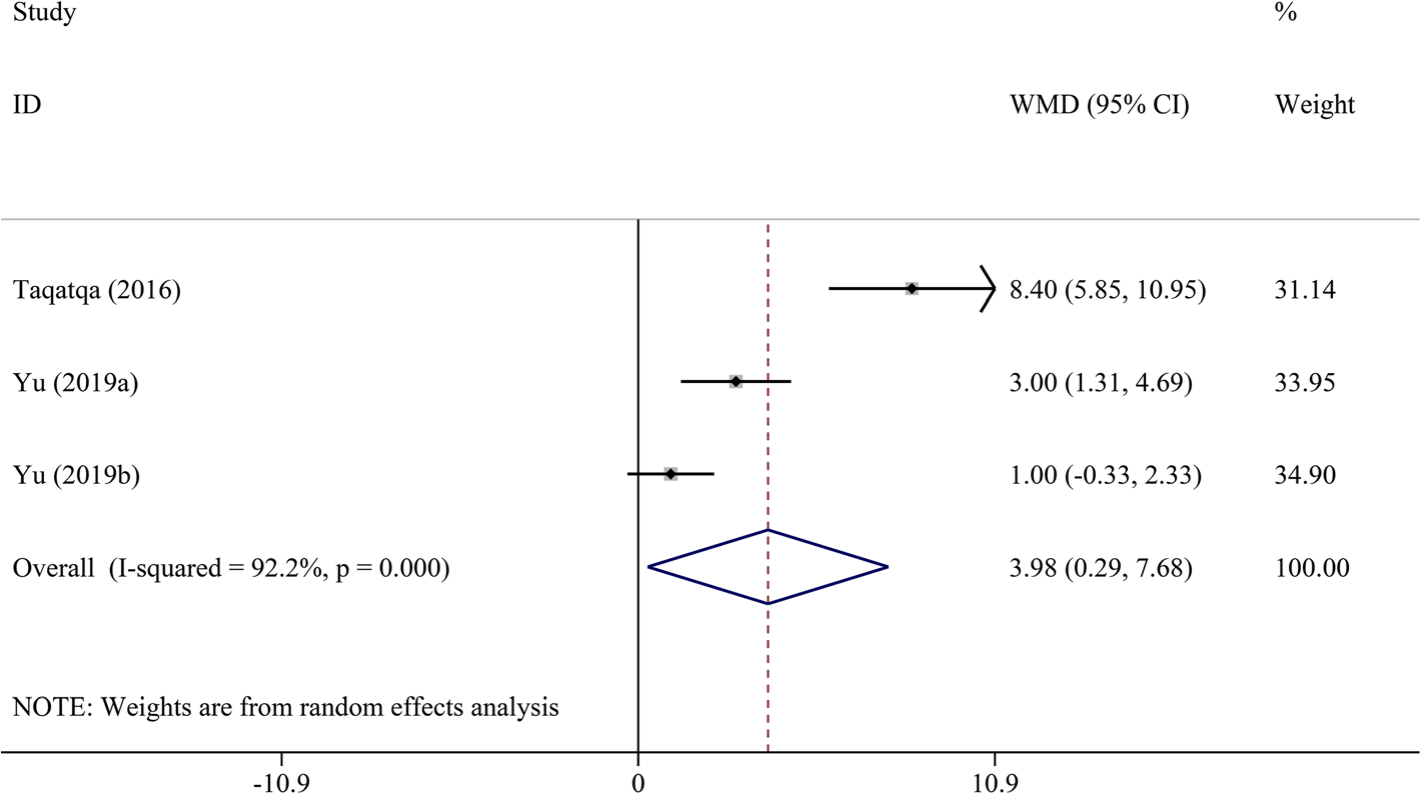
The meta-analysis of WMD in GCS between DMD and control groups. CI, confidence interval; DMD, Duchenne muscular dystrophy; GCS, global circumferential strain; WMD, weighted mean difference

**Fig. 5:**
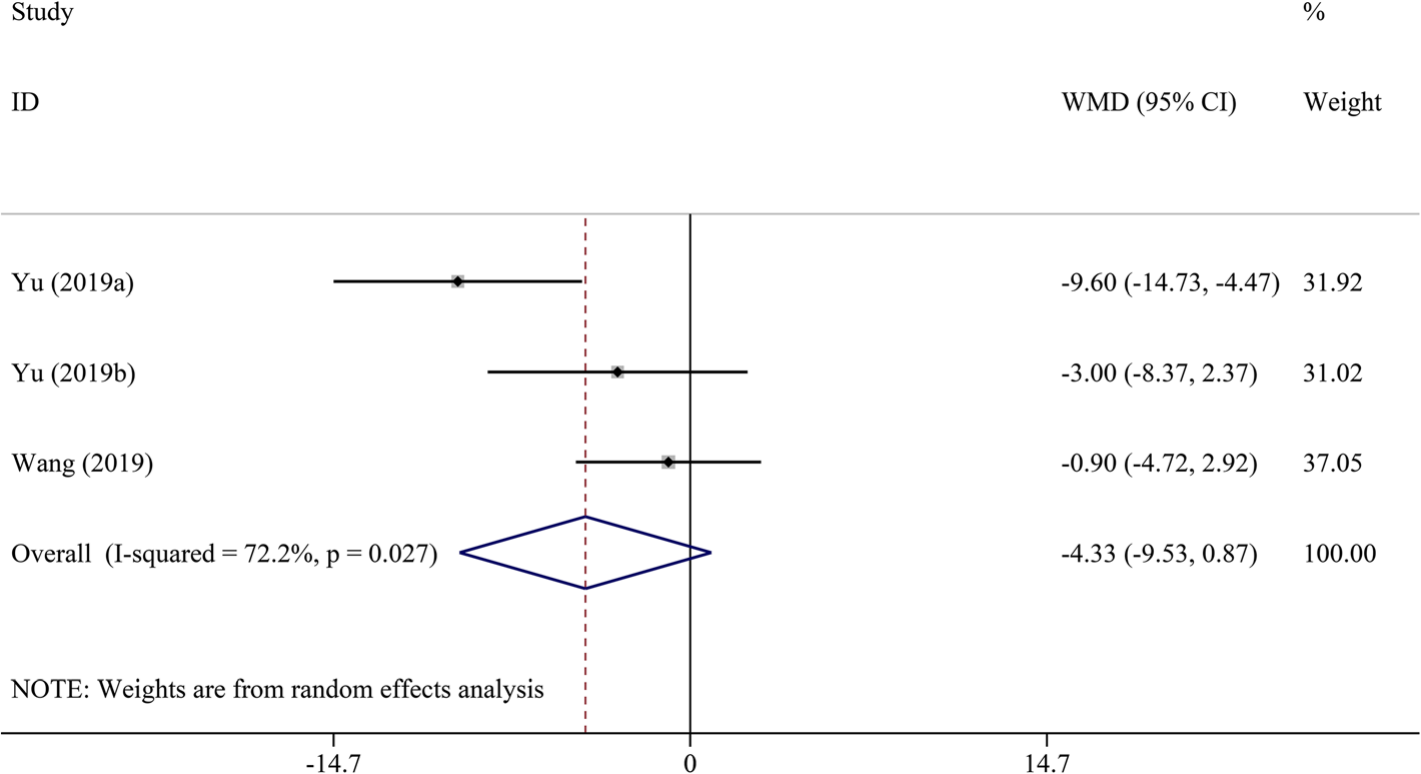
The meta-analysis of WMD in GRS between DMD and control groups. CI, confidence interval; DMD, Duchenne muscular dystrophy; GRS, global radial strain; WMD, weighted mean difference

**Fig. 6:**
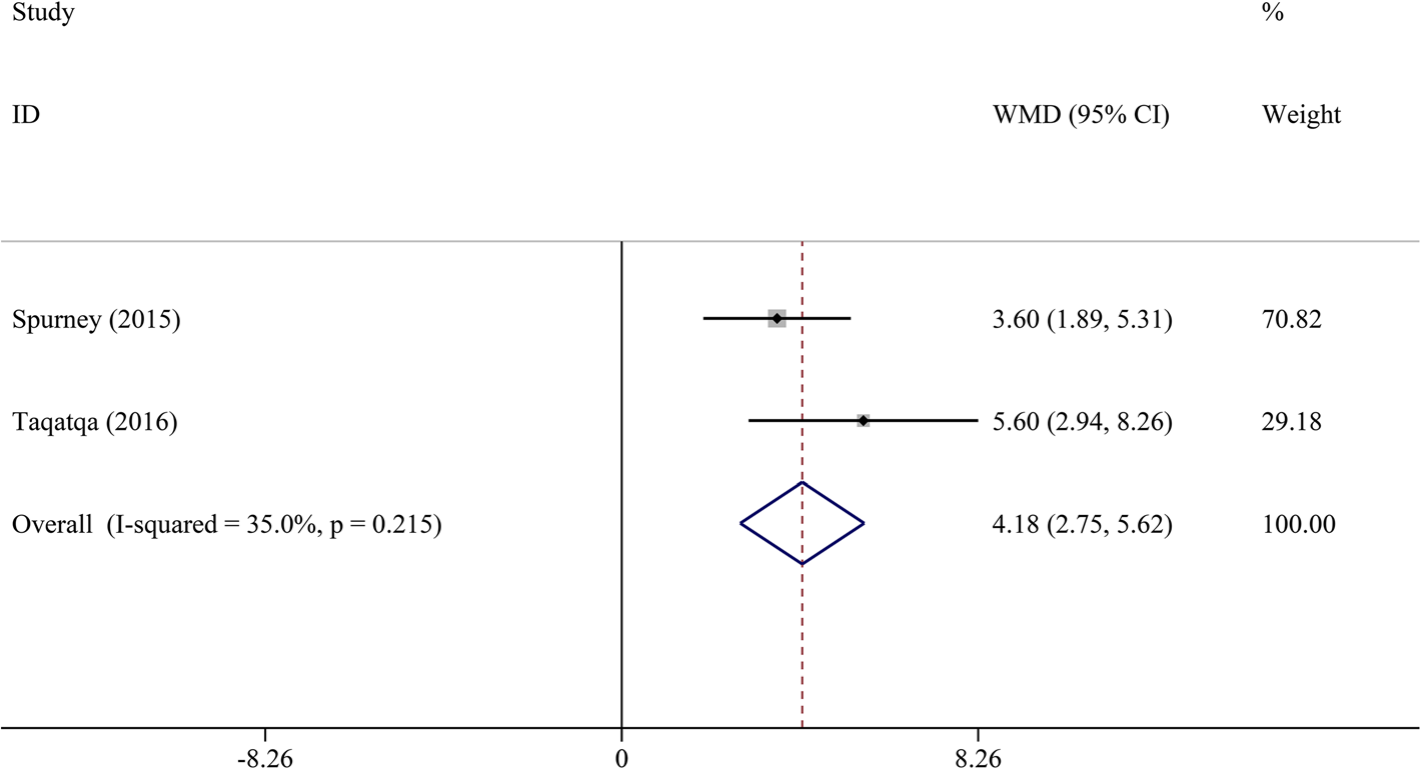
The meta-analysis of WMD in LS between DMD and control groups. CI, confidence interval; DMD, Duchenne muscular dystrophy; LS, longitudinal strain; WMD, weighted mean difference

**Fig. 7:**
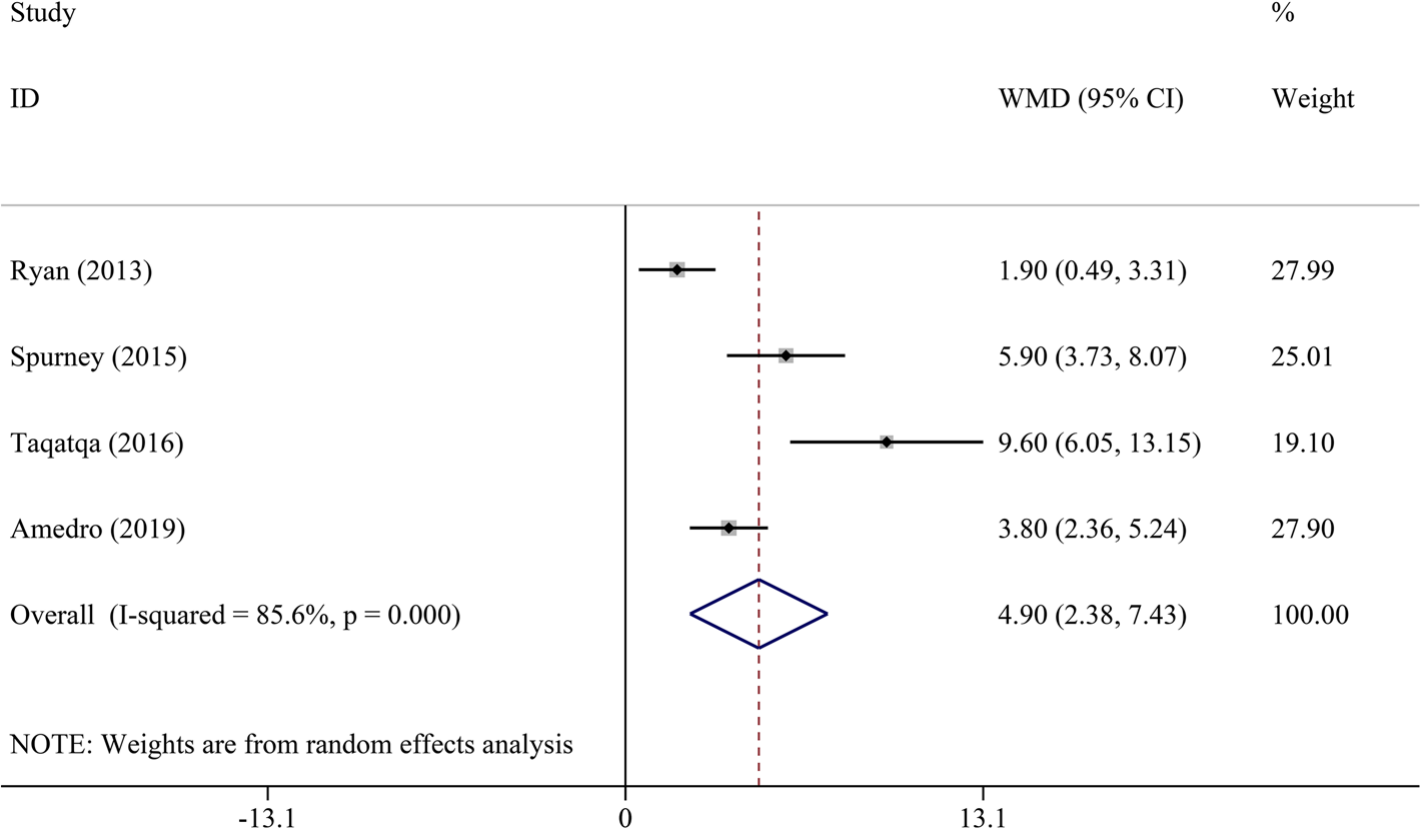
The meta-analysis of WMD in CS between DMD and control groups. CI, confidence interval; CS, circumferential strain; DMD, Duchenne muscular dystrophy; WMD, weighted mean difference

Pooled analysis indicated that LVEF was unchanged in the DMD group compared to the control group (WMD = − 1.00, 95% CI: − 2.70–0.70, *P* = 0.249) (Table 2, Fig. 2). STE revealed that GLS, GCS, LS, and CS had a significant decrease in DMD children compared to the control group (WMD = 4.17, 95% CI: 3.03–5.32; WMD = 3.98, 95% CI: 0.29–7.68; WMD = 4.18, 95% CI: 2.75–5.62; and WMD = 4.90, 95% CI: 2.38–7.43, respectively; all *P* < 0.05) (Figs. 3, 4, 5, 6 and 7). However, GRS was unchanged in the DMD group compared to the control group (WMD = − 4.33, 95% CI: − 9.53–0.87, *P* = 0.103).

**Table 2:** The meta-analysis of myocardial strain and LVEF between DMD and control groups

Parameter	Test of difference		Test of Heterogeneity		Statistical Model	Test of Publication Bias Egger’s *P-*value	Sensitivity Analysis	
	WMD (95% CI)	*P* value	*I*^2^ (%)	*P* value			Odds Ratio (95% CI) _min_	Odds Ratio (95% CI) _max_
GLS	**4.17 (3.03–5.32)**	< 0.001	62.0	0.010	Random	0.498	3.81 (2.76–4.86)	4.56 (3.75–5.36)
GCS	**3.98 (0.29–7.68)**	0.034	92.2	< 0.001	Random	0.023	1.93 (−0.23–3.88)	5.61 (0.32–10.90)
GRS	−4.33 (−9.53–0.87)	0.103	72.2	0.027	Random	0.525	−6.35 (−12.82–0.12)	−1.61 (−4.72–1.51)
LS	**4.18 (2.75–5.62)**	< 0.001	35.0	0.215	Fixed	–	3.60 (1.89–5.31)	5.60 (2.94–8.26)
CS	**4.90 (2.38–7.43)**	< 0.001	85.6	< 0.001	Random	0.085	3.74 (1.65–5.82)	6.05 (3.17–8.92)
LVEF	−1.00 (−2.70–0.70)	0.249	76.5	< 0.001	Random	0.651	−1.45 (−3.19–0.28)	−0.37 (− 1.94–1.19)

*CI* confidence interval, *CS* circumferential strain, *DMD* Duchenne muscular dystrophy, *GCS* global circumferential strain, *GLS* global longitudinal strain, *GRS* global radial strain, *LS* longitudinal strain, *LVEF* left ventricular ejection fraction, *WMD* weighted mean difference. Significant results are marked in bold

### Subgroup analysis

To investigate the possible sources of GLS heterogeneity (*I*^2^ = 62.0%, *P* = 0.010) as a main echocardiographic parameter, subgroup analysis was carried out and indicated that significant results were observed in all subgroup analyses (Table 3).

**Table 3:** Subgroup analyses of GLS between DMD and control groups

	Number of studies	Test of difference		Test of Heterogeneity	
		WMD (95% CI)	*P* value	*I*^2^ (%)	*P* value
Country					
USA/Europe/Egypt	4	5.01 (3.73–6.29)	< 0.001	40.5	0.169
China/Korea	4	3.28 (1.63–4.93)	< 0.001	62.4	0.047
Quality of study					
Medium quality	5	4.89 (3.78–6.00)	< 0.001	28.3	0.233
High quality	3	3.04 (1.04–5.05)	0.003	71.5	0.030
Method					
Two-dimensional STE	4	4.90 (3.62–6.19)	< 0.001	40.1	0.171
Three-dimensional STE	4	3.41 (1.64–5.17)	< 0.001	67.8	0.025
Machine/software					
GE Vivid E9 with EchoPAC	7	4.07 (2.83–5.31)	< 0.001	66.1	0.007
Philips iE33 with Qlab	1	5.20 (2.51–7.89)	< 0.001	–	–

*CI* confidence interval, *GLS* global longitudinal strain, *STE* speckle tracking echocardiography, *WMD* weighted mean difference

### Sensitivity analysis and publication bias

To evaluate the robustness of the results, sensitivity analyses were performed by sequentially removing each study. As a result, no apparent change occurred in the GLS, LS, CS, and LVEF when an individual study was omitted, confirming that the results were stable (Table 2). No publication bias was detected among studies focused on the GLS, GRS, or LVEF, which was confirmed by the Egger’s tests (Table 2).

### Trial sequential analysis

**Fig. 8:**
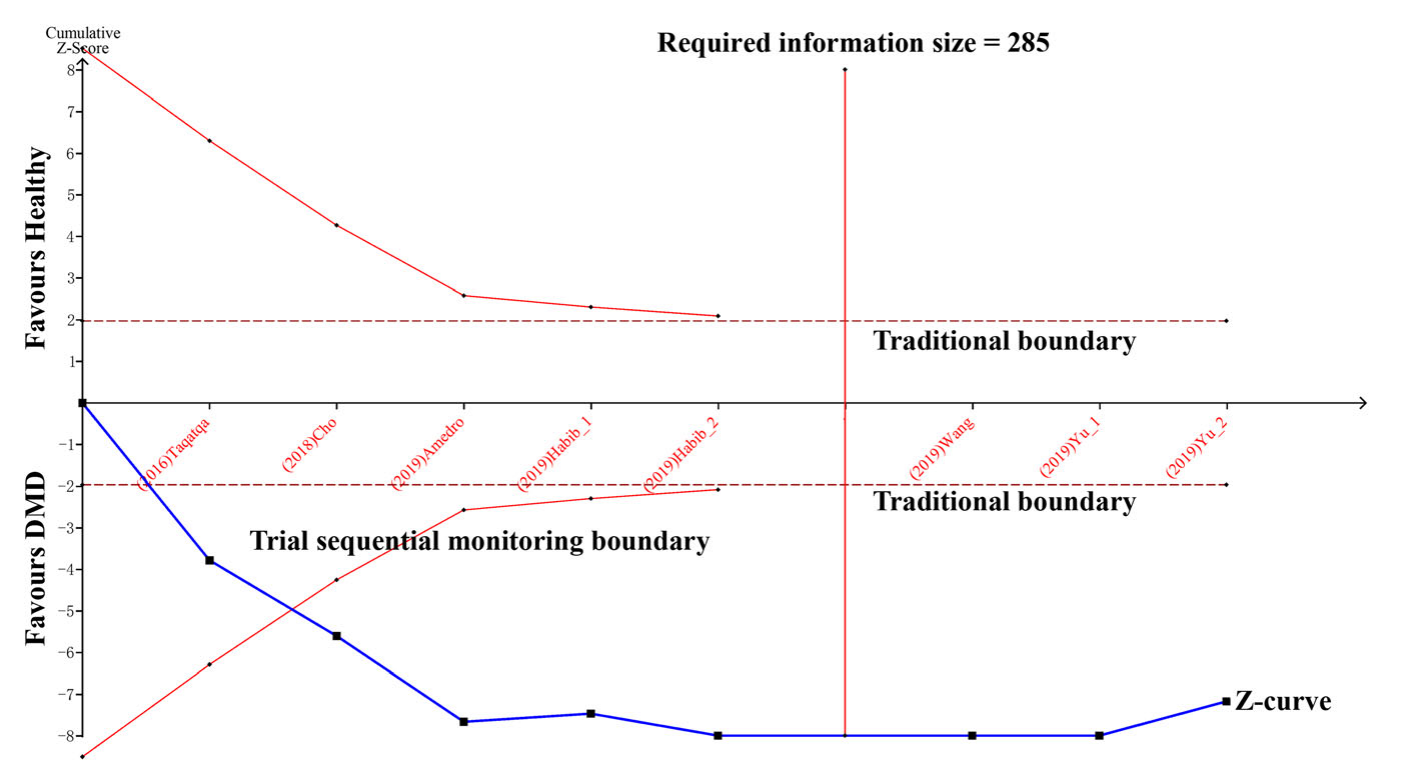
Comparison of the GLS between DMD and control groups by TSA. DMD, Duchenne muscular dystrophy; GLS, global longitudinal strain; TSA, trial sequential analysis

The cumulative Z-curve for GLS, LS, and CS passed both the traditional boundary and trial sequential monitoring boundary, suggesting sufficient evidence for such a difference between the DMD and control groups (Fig. 8, and Figure S1-S2 in Supplement file).

The cumulative Z-curve for GCS crossed the traditional boundary, but GRS did not. Neither GCS nor GRS reached the trial sequential monitoring boundary or required information size line, suggesting that more studies are needed for further analysis (Figure S3-S4 in Supplement file).

## Discussion

This is the first meta-analysis comprehensively summarizing the early changes in myocardial strain in DMD children. In this meta-analysis, GLS served as an explicit index for early detection, which was confirmed by sensitivity analysis, publication bias test, and TSA. CS and LS were affected by the small number of involved studies, which can decrease their certainty. TSA indicated that GCS and GRS needed more research data to reach a firm conclusion.

Most DMD patients develop cardiomyopathy by the age of 20 years [21]. Some DMD children with cardiac involvement were undertreated or not being treated with cardiac-specific medicines in clinical practice [4]. Although DMD cannot be cured, some medicines may modify the course of cardiomyopathy and be beneficial for delaying progression of heart failure [22–24]. The onset of symptoms and reduced LVEF were the important factors affecting the timing of medical treatment [5, 6]. Unfortunately, the patients were often beyond the point of reversible injury [25]. The reasons were the following: (i) cardiac dysfunction is progressing very slightly and limited daily activities in DMD children may cover up their initial symptoms; (ii) as the most common index to assess left ventricle contraction, LVEF usually fails to detect early cardiac systolic dysfunction [26]. Hence, detection of early pre-symptomatic cardiac involvement using a new index in DMD children is urgently needed.

Before symptoms appeared and LVEF decreased, the myocardial strain has emerged as a promising parameter of subclinical myocardial dysfunction. A recent review stated that myocardial strain can be assessed via cardiac magnetic resonance or echocardiography [27]. Cardiac magnetic resonance has been used to measure strain. However, it may be challenging in patients with DMD, especially in young children who cannot cooperate during the examination [28]. In the past, echocardiographers measured myocardial strain using tissue Doppler imaging, which is limited by poor echocardiographic windows and the probe angle [27]. STE is the preferred technique for measuring myocardial strain to provide a rapid, precise, and objective assessment of the left heart function [29, 30]. The high repeatability of STE is due to its independence on the angle, which allows to measure only the active contraction, avoiding the tethering effect of noncontractile tissue.

STE can assess the global or segmental myocardial strain. However, segmental strain in DMD children has high variability. For segmental longitudinal strain, Mertens et al. study revealed that anterolateral segmental longitudinal strain is more severely impaired [31]. Taqatqa et al. showed that apical segmental longitudinal strain is more pronounced [15]. Furthermore, Cho et al. found that basal segmental longitudinal strain is serious [16]. Finally, Amedro et al. revealed that segmental longitudinal strain in most segments decreased in DMD children [18]. Therefore, meta-analysis in the present study focused on the global/average myocardial strain. In these five types of strain, GLS provided the best evidence for early detection of myocardial strain in DMD children based on the existing data. This ability to detect subclinical myocardial dysfunction in patients with normal LVEF was consistent with other research findings [32, 33]. According to the result by Yu et al., GLS has an 82.1% sensitivity to identify DMD when using − 20.5% as a cutoff value [17]. GLS did not only detect this subtle left ventricular systolic dysfunction, but also played a key role in detecting other cardiomyopathies [34, 35]. The 3D and 2D global strain values correlated well in both normally and abnormally contracting hearts in children [36–38]. However, stratified analysis using echocardiography methods was still used, considering the possible differences arising from the inclusion of both 2D and 3D strains in DMD children. No differences were present between 3D and 2D GLS subgroups. GLS measured by STE has its own limitation, as the quality of the echocardiographic images decreases with increasing age in DMD children and young adults [39]. Furthermore, the lack of GLS accepted normal reference also limited its further clinical applications.

There are several limitations in this study. First, older children (> 8 years old) had a lower GLS than younger children (≤ 8 years old) [17]. However, the data were not sufficient to perform subgroup analysis by age in this meta-analysis. Second, global area strain was not analyzed in this meta-analysis due to insufficient sample size.

## Conclusion

GLS can be useful for early detection of left ventricle myocardial dysfunction in children with DMD. The role of other myocardial strains in DMD children needs further study.

## Supplementary information

**Additional file 1: Figure S1.** Comparison of the LS between DMD and control groups by TSA. DMD, Duchenne muscular dystrophy; LS, longitudinal strain; TSA, trial sequential analysis. **Figure S2** Comparison of the CS between DMD and control groups by TSA. CS, circumferential strain; DMD, Duchenne muscular dystrophy; TSA, trial sequential analysis. **Figure S3** Comparison of the GCS between DMD and control groups by TSA. DMD, Duchenne muscular dystrophy; GCS, global circumferential strain; TSA, trial sequential analysis. **Figure S4** Comparison of the GRS between DMD and control groups by TSA. DMD, Duchenne muscular dystrophy; GRS, global radial strain; TSA, trial sequential analysis.

## Data Availability

The datasets used and/or analyzed during the current study are available from the corresponding author on reasonable request.

## Abbreviations

CIs: Confidence intervals
CS: Circumferential strain
DMD: Duchenne muscular dystrophy
GCS: Global circumferential strain
GLS: Global longitudinal strain
GRS: Global radial strain
LS: Longitudinal strain
LVEF: Left ventricular ejection fraction
NOS: Newcastle–Ottawa scale
STE: Speckle-tracking echocardiography
TSA: Trial sequential analysis
WMD: Weighted mean difference
