# Supplementary material for: Usefulness of speckle-tracking echocardiography for early detection in children with Duchenne muscular dystrophy: a meta-analysis and trial sequential analysis
Source: Cardiovasc Ultrasound. 2020 Jul 10;18:26. doi: 10.1186/s12947-020-00209-y (PMC7353706; doi:10.1186/s12947-020-00209-y)
Supplement: Supplementary file 1 — Additional file 1: Figure S1. Comparison of the LS between DMD and control groups by TSA. DMD, Duchenne muscular dystrophy; LS, longitudinal strain; TSA, trial sequential analysis. Figure S2 Comparison of the CS between DMD and control groups by TSA. CS, circumferential strain; DMD, Duchenne muscular dystrophy; TSA, trial sequential analysis. Figure S3 Comparison of the GCS between DMD and control groups by TSA. DMD, Duchenne muscular dystrophy; GCS, global circumferential strain; TSA, trial sequential analysis. Figure S4 Comparison of the GRS between DMD and control groups by TSA. DMD, Duchenne muscular dystrophy; GRS, global radial strain; TSA, trial sequential analysis. [file 12947_2020_209_MOESM1_ESM.pdf]

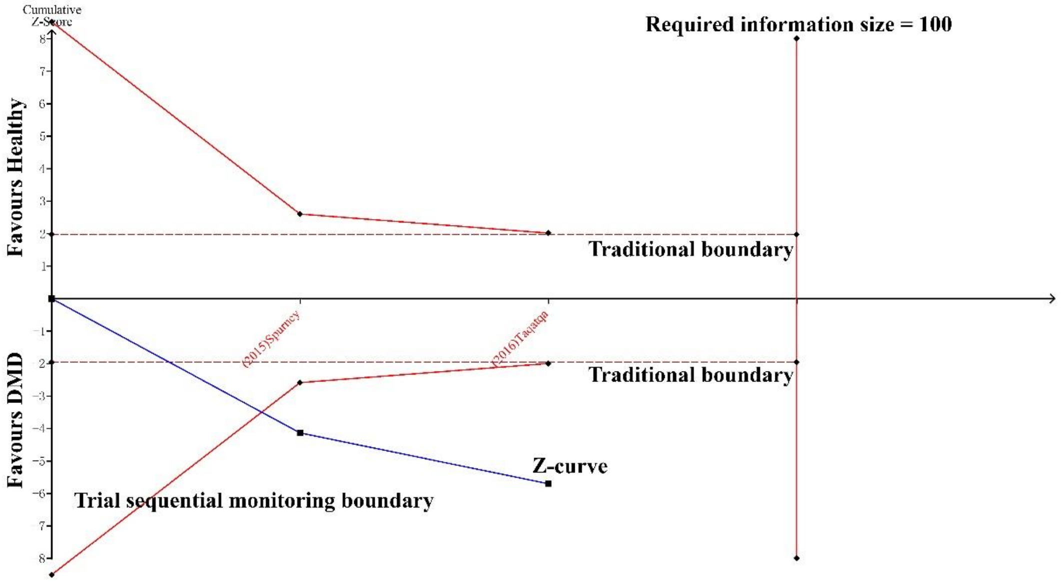

Figure S1 Comparison of the LS between DMD and control groups by TSA. DMD, Duchenne muscular dystrophy; LS, longitudinal strain; TSA, trial sequential analysis.

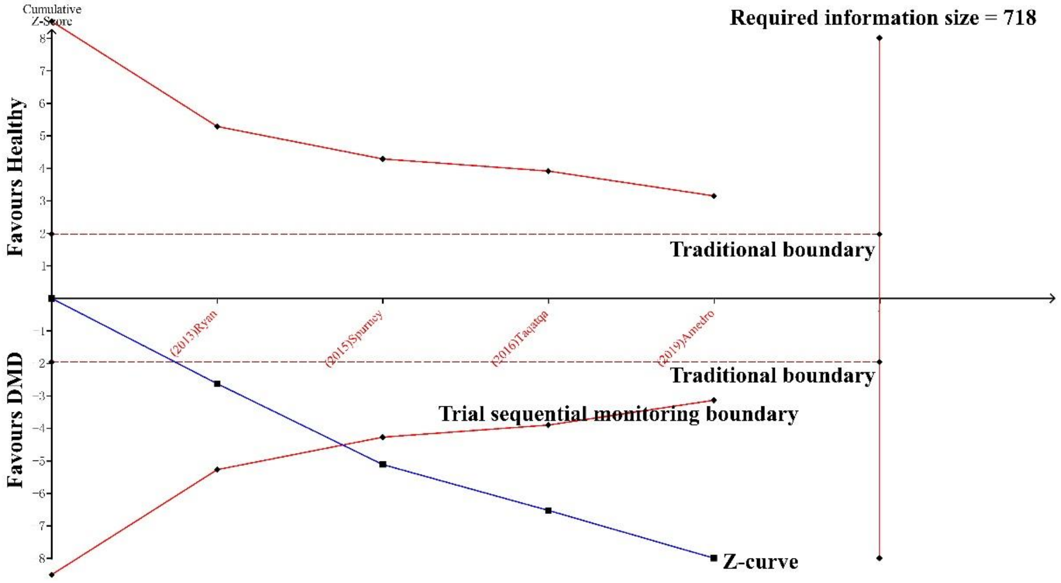

Figure S2 Comparison of the CS between DMD and control groups by TSA. CS, circumferential strain; DMD, Duchenne muscular dystrophy; TSA, trial sequential analysis.

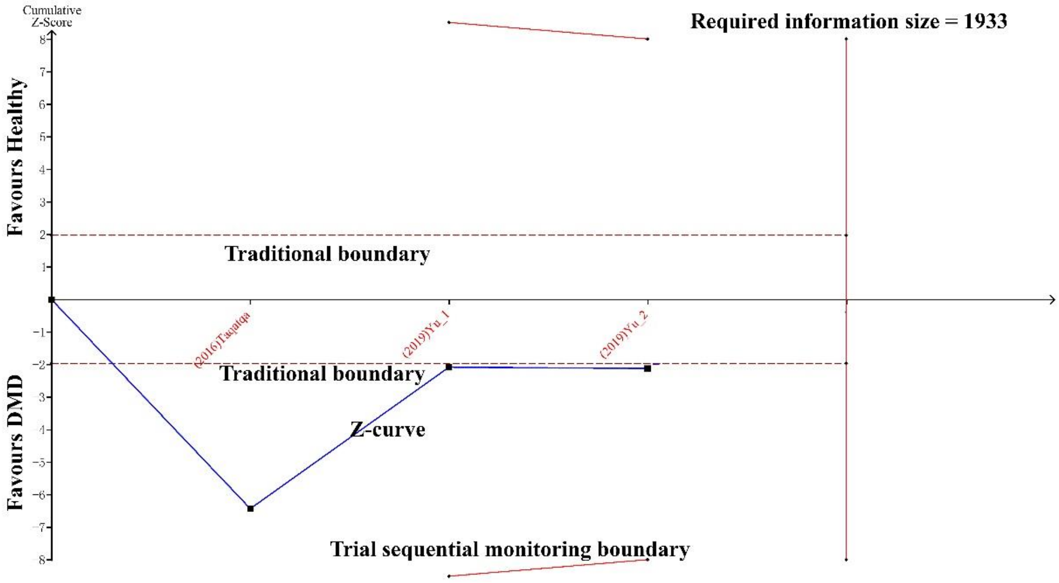

Figure S3 Comparison of the GCS between DMD and control groups by TSA. DMD, Duchenne muscular dystrophy; GCS, global circumferential strain; TSA, trial sequential analysis.

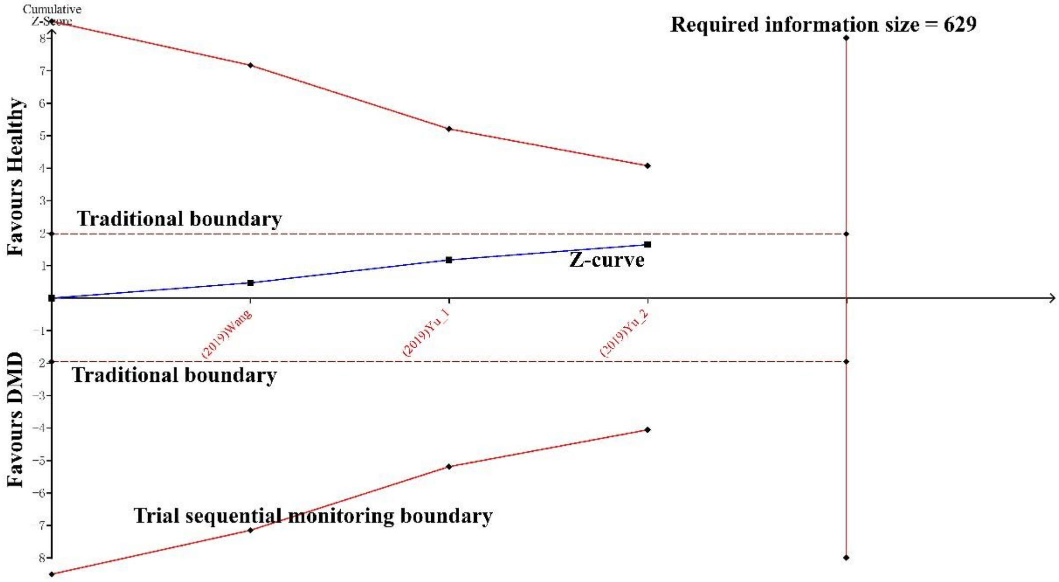

Figure S4 Comparison of the GRS between DMD and control groups by TSA. DMD, Duchenne muscular dystrophy; GRS, global radial strain; TSA, trial sequential analysis.
